# Draft Genome Sequences of Two Strains of *Salmonella enterica* Serovar Typhimurium Displaying Different Virulence in an Experimental Chicken Model

**DOI:** 10.1128/genomeA.01526-16

**Published:** 2017-02-09

**Authors:** Dele Ogunremi, Burton Blais, Hongsheng Huang, Linru Wang, Mohamed Elmufti, Ray Allain, Jennifer Hazelwood, Chris Grenier, Kingsley Amoako, Mirjana Savic, Nashmil Fattahi Ghazi

**Affiliations:** a Ottawa Laboratory Fallowfield, Canadian Food Inspection Agency, Ottawa, Ontario, Canada; b Ottawa Laboratory Carling, Canadian Food Inspection Agency, Ottawa, Ontario, Canada; c Greater Toronto Area Laboratory, Canadian Food Inspection Agency, Scarborough, Ontario, Canada; d Food Safety Services Division, Canadian Food Inspection Agency, Ottawa, Ontario, Canada; e Lethbridge Laboratory, Canadian Food Inspection Agency, Lethbridge, Alberta, Canada

## Abstract

*Salmonella enterica* serovar Typhimurium strains 22495 and 22792, obtained from wild birds, were found to display different virulence attributes in an experimental chicken model. Closed genome sequences were assembled after sequencing with the Roche 454 and Illumina MiSeq platforms. An additional plasmid was present in the more virulent strain 22495.

## GENOME ANNOUNCEMENT

*Salmonella enterica* serovar Typhimurium is a common foodborne bacterial pathogen causing gastroenteritis and sometimes disseminated, fatal infection in immunocompromised individuals. During a study using an experimental chicken infection model to assess the virulence of *S*. Typhimurium among a collection from wild birds (1), we observed two strains of *S*. Typhimurium with different virulence. One *S*. Typhimurium isolate, recovered from a wild cormorant in Saskatchewan, Canada, in 2008 and designated strain 22972 phage type 1, was shown to cause a low mortality in day-old chicks (16%; *n* = 25). In comparison, *S*. Typhimurium 22495 phage type 41, which was obtained from a wild sea gull in Saskatchewan, Canada, in 2008 was more virulent (50% mortality, *n* = 18). Pulsed-field gel electrophoresis analysis showed that the more virulent strain 22495 has a molecular fingerprinting pattern designated XAI 0269/BNI 0081. A search of the Canadian PulseNet database showed that the fingerprint matched clinical isolates from foodborne cases involving 30 people from seven Canadian provinces—namely, Alberta, British Columbia, Nova Scotia, Ontario, Prince Edward Island, Quebec, and Saskatchewan. Four clusters of foodborne illness cases involving three to five provinces in each outbreak were detected starting from the year 2004, when the first clinical case was documented by PulseNET, up to the present (2017). The less virulent strain 22792 had a fingerprint designation, XAI 0654/BNI 0295, which, other than this particular instance in a cormorant, has not previously been found in Canada.

We carried out genome sequencing of the two strains using alternate sequencing chemistry on the Roche 454 (Roche Diagnostics, Mannheim, Germany) and Illumina MiSeq (Illumina, San Diego, CA, USA) platforms. Raw reads from Roche 454 were assembled into scaffolds by use of the Newbler assemblers, while the Illumina raw reads were assembled into contigs using CLC Genomics Workbench version 7 (Qiagen, Waltham, MA, USA). The independently assembled contigs and scaffolds were compared to the genome (optical) map generated for each strain by means of the Argus optical mapping system (OpGen, Gaithersburg, MD, USA), leading to the assembly of scaffolds and contigs into a single chromosome for each strain. Assembled molecules that did not align to the chromosome optical map were shown to be plasmids by means of BLAST analysis. Strain 22792 has a chromosome with a size of 4,806,964 bp and a large *Salmonella* virulence plasmid of 93,595 bp. Strain 22945 has a similar-sized chromosome of 4,817,416 bp, a virulence plasmid of 93,055 bp, and a smaller plasmid of 2,097 bp. Evaluation of the genomes by means of the prokaryotic annotation software Prokka (2) revealed that strain 22495 had 4,649 coding sequences (CDSs), 75 tRNA molecules, and CRISPR repeat sequences, while strain 22792 had 4,543 CDSs, 76 transfer RNAs, and CRISPR repeat sequences. Each chromosome contains a full complement of seven ribosomal DNA operons.

### Accession number(s).

The nucleotide sequences for the chromosome, large virulent plasmid, and small plasmid for *S*. Typhimurium strain 22495 have been deposited in GenBank under accession numbers CP017617, CP017618, and CP017619, respectively, while the chromosome and large virulent plasmid for *S*. Typhimurium strain 22792 have nucleotide sequences deposited in GenBank under accession numbers CP017621 and CP017620, respectively.
